# Host-associated Genetic Import in *Campylobacter jejuni*

**DOI:** 10.3201/eid1302.060620

**Published:** 2007-02

**Authors:** Noel D. McCarthy, Frances M. Colles, Kate E. Dingle, Mary C. Bagnall, Georgina Manning, Martin C.J. Maiden, Daniel Falush

**Affiliations:** * University of Oxford, Oxford, United Kingdom; † Veterinary Laboratories Agency, Weybridge, United Kingdom

**Keywords:** Bacterial typing techniques, epidemiology, population genetics, population dynamics, zoonoses, Campylobacter jejuni, public health, disease reservoirs, ecology, communicable disease control, research

## Abstract

Establishing sources of human infection supports effective disease control measures. Host association with *Campylobacter jejuni* was analyzed by using multilocus sequence typing data for 713 isolates from chickens and bovids (cattle and sheep). Commonly used summary measures of genotypes (sequence type and clonal complex) showed poor accuracy, but a method using the full allelic profile showed 80% accuracy in distinguishing isolates from these 2 host groups. We explored the biologic basis of more accurate results with allelic profiles. Strains isolated from specific hosts have imported a substantial number of alleles while circulating in those host species. These results imply that 1) although *Campylobacter* moves frequently between hosts, most transmission is within species, and 2) lineages can acquire a host signature and potentially adapt to the host through recombination. Assignment using this signature enables improved prediction of source for pathogens that undergo frequent genetic recombination.

## Abstract 2

*C*. *jejuni* genomes have a host signature that enables attribution of isolates to animal sources.

Many human pathogens inhabit several animal host and environmental reservoirs, and a broad host range is particularly characteristic of emerging diseases (*1*). Identification of the relative contributions of pathogen sources and transmission routes is necessary to support evidence-based disease control programs (*2*). One approach to this identification, microbial source tracking, is the application of microbial typing to isolates from human cases and possible sources in the food chain to enable attribution of disease to food sources at individual case and population levels (*3*,*4*). Evidence-based control programs using this information have provided accurate results for *Salmonella* at a population level in Denmark (*4*).

Source tracking depends on accurate estimation of the frequency of different subtypes in each host reservoir. For *Salmonella*, specific serotypes and phage subtypes are stably found in the same host (*3*). The biology underlying this success is first that specific clones are well-adapted to specific hosts and second that the combination of serotype and phage type provides a stable and reliable indicator of a specific clone. For other organisms it can be difficult to find reliable host-associated markers. One example is *Campylobacter jejuni*, the most common zoonosis and the main cause of bacterial gastroenteritis in the western world. However, phenotyping has not worked well in determining source. Genetic methods of discrimination show large diversity of results within this species; studies typically report ≈50% as many genotypes as strains (*5*–*12*). Many common genotypes are broadly distributed and it is not possible to estimate the relative frequency of genotypes in different host reservoirs accurately. Because of these difficulties, although host associations have been identified for particular genotypes, no generally useable approach has been developed.

We developed an approach that uses multilocus sequence typing (MLST) data to identify the reservoir of origin of a strain. This approach was tested by using isolates from known sources (cattle, sheep, and chickens), which allowed us to compare our predictions with the true origin of each strain. This method can provide reasonably accurate results for rare or unique genotypes and for clones that are broadly distributed. This approach takes into account frequent recombination in *Campylobacter*, which limits the accuracy of approaches based on the *Salmonella* paradigm.

## Methods

### Data

MLST of *C*. *jejuni* is based on sequencing 7 loci with lengths of 402–507 bp separated from each other by >15,000 bp in the type strain (*10*). We used MLST data in 3 different forms. The first form was the sequence type (ST), which is a unique combination of 7 alleles. STs index the full discrimination available within MLST. The second form was the clonal complex, which is a group of closely related STs, e.g., differing at >2 of the 7 alleles. Clonal complexes, if accurately inferred, are groups of strains that share a more recent common ancestor than with strains outside the complex but are not identical to each other at all of the MLST loci (*10*,*13*,*14*). The third form was the 7 allele fragments; we assumed that they each provided independent information.

We included all *C*. *jejuni* isolates from cattle, sheep and chickens that were in the pubmlst database (www.pubmlst.org) with a date before August 1, 2004, and which had been published in peer-reviewed literature or for which permission to use in this study was obtained from those who had submitted the data. All but 10 of the isolates on pubmlst were available for inclusion by these criteria. We also included additional typed isolates (n = 27) provided by researchers when they were contacted for permission to include unpublished isolates from the pubmlst database. *C*. *jejuni* has been shown to recombine with *C*. *coli* (*15*). Those isolates with >4 of 7 alleles typical of *C*. *jejuni* were included. A total of 713 isolates were available by these criteria and came from animal feces, live animals, and dead animal tissue. The distribution of the data by host type and by year and country of isolation is shown in Tables 1 and 2.

**Table 1:** *Campylobacter jejuni* isolates by year of isolation and host species

Year	Chickens	Cattle	Sheep	Total
1981	0	4	0	4
1982	2	1	2	5
1983	0	3	0	3
1984	0	1	0	1
1986	0	2	0	2
1988	2	18	0	20
1989	0	1	0	1
1990	54	1	0	55
1991	30	6	0	36
1992	1	3	0	4
1993	8	6	1	15
1994	6	1	0	7
1995	12	1	0	13
1996	35	0	0	35
1997	2	0	0	2
1998	40	41	68	149
1999	10	38	38	86
2000	15	6	0	21
2001	45	83	5	133
2002	0	0	2	2
2003	13	0	0	13
Unspecified	34	29	43	106
Total	309	245	159	713

**Table 2:** *Campylobacter jejuni* isolates by country and host species

Country	Chicken	Cattle	Sheep	Total
Canada	0	5	0	5
Czech Republic	8	0	0	8
Denmark	6	1	0	7
The Netherlands	53	4	0	57
New Zealand	5	1	0	6
Northern Ireland	1	2	0	3
United Kingdom	217	218	158	593
United States	17	13	1	31
Unknown	2	1	0	3
Total	309	245	159	713

### Population Assignment

Differences in genotype frequency between populations enable probabilistic assignment of isolates to populations, even if some sharing of genotypes occurs between those populations. We used STRUCTURE, a model-based clustering method designed to infer population structure and assign individuals to populations by using multilocus genotype data (*16*). The source of the isolates to be assigned was predicted on the basis of a training set that consisted of other relevant isolates. To do this predicting, we used the USEPOPINFO option, which allows the population of origin to be known for some strains (in this instance, the training set) while for other strains (the isolates to be assigned) this population is assumed unknown.

STRUCTURE estimates the genotype frequencies in each host species based on all of the isolates; it also estimates the population of origin for isolates of unknown origin, taking into account uncertainty due to sample size. To enable maximum use of data, some analyses used a leave-one-out strategy in which 1 isolate was assigned by using the remaining strains as the training dataset and the procedure was repeated for each isolate.

The parameters we used for all STRUCTURE simulations were a no-admixture model with λ = 1 and gene frequencies uncorrelated between populations. We ran 1,000 burn-in cycles and 10,000 additional repetitions for each analysis. Empiric assignment accuracy was measured as the average probability pk* with which each isolate was assigned to the correct host source k*. Predicted assignment accuracy is estimated as the average of , where each individual is assigned to 1 of *K* different sources. The permutation test (Figure, panel A) was performed by randomly permuting the actual host species among the predictions obtained from STRUCTURE repeated 10,000 times.

## Results

Among 713 isolates, 330 MLST genotypes were identified. Two isolates (ST-284 and ST-327) had 4 alleles typical of *C*. *jejuni* and 3 typical of *C*. *coli*. All others had >5 typical *C*. *jejuni* alleles. Table 3 shows assignment accuracy when we used the whole dataset and a leave-one-out strategy to assign strains to 3 host species (cow, sheep, and chicken) on the basis of 7 alleles, the clonal complex, the ST, and combinations thereof. Because random assignment would be correct one third of the time, how much improvement genotype information showed compared with random assignment is more informative than the percentage correct, i.e., what proportion of the gap between 33% correct expected by using random assignment and 100% correct with perfect prediction has been closed. Assignment by using the 7 alleles closed 37% of this gap compared with 10% for ST and 13% for clonal complex. Prediction did not improve substantially when ST or clonal complex information was added to allele information. These overall results emphasize the limits in using an ST or clonal complex as a summary of MLST when predicting host of origin. We therefore used alleles in all further analyses and explored the basis for the better accuracy of this approach.

**Table 3:** Capacity of alleles, overall sequence type, and clonal complex information to predict host species for *Campylobacter jejuni* isolates from cattle, sheep, and chickens

Genotype information used	% correct	% uncertainty removed*
Alleles	58	37
Sequence type	40	10
Clonal complex (1)†	42	13
Clonal complex (2)†	42	13
Alleles plus sequence type	60	40
Alleles plus clonal complex†	58	37

*Random selection would be expected to predict correctly 33% of the time. The proportion of the remaining uncertainty (67%) that is resolved is shown.

†Clonal complex (1) substituted sequence type for clonal complex in which no clonal complex is assigned and clonal complex (2) substituted a missing value code. Clonal complex (1) was also used to assess alleles plus clonal complex.

Prediction of host of origin to 3 host sources on the basis of alleles is shown in Table 4. The method showed higher accuracy for distinguishing chicken strains from cow or sheep strains than for distinguishing between strains from the 2 bovid species. When we performed analysis restricted to cattle and sheep isolates, we obtained an assignment accuracy of 58% compared with 50% expected by chance and thus explained only 16% of remaining uncertainty. This additional analysis showed little detectable host association for these 2 closely related host species. Further comparison of chicken isolates with a combined population from cattle and sheep showed improved resolution and allowed correct prediction 80% of the time (60% of uncertainty removed), which indicated substantial host association.

**Table 4:** Comparison of actual host and predicted host among *Campylobacter jejuni* from cattle, sheep, and chickens

Actual host	Sample size (n)	Predicted host, %		
		Chicken	Cow	Sheep
Chicken	309	66	14	19
Cow	245	12	50	38
Sheep	159	10	36	54

Given the nature of the dataset, we must consider possible confounding factors such as differences in time or location of sampling, which may lead either to completely spurious associations or to overestimates of their magnitude. Indeed, there was evidence for modest time and geographic effects within our dataset. For example, in a comparison of UK chicken isolates in 1997 or earlier and in 1998 or later (Table 5), 66% could be assigned to the population of the correct period based on allelic profile. Similarly, when UK and Dutch chicken isolates were considered, 69% were assigned to the correct country. We therefore performed additional analyses in which host was negatively associated with time, space, or both (Table 5). Late UK chicken isolates (1998–2003) were assigned by using early UK chicken (1997 or earlier) and late UK bovid isolates (1998–2003) as training sets, which indicated 77% assignment to chickens. UK chicken isolates were assigned by using non-UK chickens and UK bovid isolates as training sets, which indicated 64% assignment to chickens. These analyses showed that host effect is stronger than that of time or space and that our findings are not the result of confounding by these factors.

**Table 5:** Animal subpopulations used to study the effect of time and sample location on isolates of *Campylobacter jejuni*

Source*	No. animals
Early UK chickens	114
Late UK chickens	78
All UK chickens	217
Dutch chickens	53
Non-UK chickens	92
Late UK cattle and sheep	273

*Early, 1990–1997; late, 1998–2003.

To explore the mechanism underlying the better performance observed for allele-based assignment and to better understand the biologic processes that produce this host signature in the bacterial genome, we investigated assignment within the ST-21 complex. This clonal complex comprises a substantial proportion of isolates and is highly diverse (*5*,*10*,*17*,*18*). Our sample contained 252 ST-21 complex isolates. Of these, 188 were not ST-21 but differed at 1 to 3 alleles from the central genotype. We assigned these 188 isolates to chicken or bovid hosts on the basis of only alleles at which they differed from ST-21 by using all non–ST-21 complex isolates as the training set. A total of 66% of isolates were assigned to the correct host. This finding suggests that ST-21 complex isolates acquire alleles that are characteristic of the host population. To demonstrate that this deviation from 50% is not a sampling artifact or chance effect, we restricted analysis to the 88 unique ST-host combinations, which largely eliminates the possible effects of clonal expansion within host, and performed a permutation test to assess the possible role of chance. Of these combinations, 67% were correctly assigned, which was a higher proportion than observed in any of 10,000 iterations in a permutation test (Figure, panel A).

The overall accuracy of host assignment based on acquired alleles is limited because many of these alleles are each too rare to enable accurate estimation of their frequency in particular host gene pools. Acquired alleles that are frequently observed give more accurate host prediction. To illustrate this visually (Figure, panel B), we used as predictors only those alleles that are found in >10 different ST-host combinations in the non–ST-21 complex isolates and substantially differentiated between chicken and bovid populations (on the basis of a 65% cut-off value). All 4 isolates with 2 alleles, both suggestive of either chicken or bovid origin, were from the predicted source. In 1 instance, 2 potentially informative alleles gave conflicting information; 1 suggested bovid origin and 1 suggested chicken origin. Isolates with this ST came from both sources. Of the 24 STs with only 1 informative allele, 18 were correctly assigned; only 4 were incorrectly assigned. The remaining 2 STs were isolated from chicken and bovid sources.

## Discussion

Our analyses confirm the association of *C*. *jejuni* genotypes with host species, and demonstrate a clear distinction between isolates obtained from chickens and those obtained from bovids, when alleles are considered independently in statistical analysis. This finding was robust to sampling differences in time and place and suggested that host effects were stronger than geographic and temporal effects, which must be considered if these associations are to be used in epidemiologic investigations. Moreover, because populations of *C*. *jejuni* in farm animals such as bovids and chickens may show greater similarity than those from other hosts (*5*,*9*), the approach may be more accurate when considering *C*. *jejuni* from a more diverse host range. The distinction between cow and sheep isolates is much weaker. Differentiation between these species might be demonstrable if more genetic information was available. However, the minor differences observed may be a sampling artifact with these species sharing a common gene pool.

The allele-based method we have used helps alleviate the problem of excess discrimination in *Campylobacter* typing. Many alleles show differences in frequency between hosts. These alleles provide useful information on source for STs that are too rare to allow estimates of their frequency in different hosts (e.g., because they are absent from training sets).

Our approach has some limitations that must be considered in any more extensive application. The current accuracy estimate of 80% in distinguishing chicken isolates from bovid ones may be optimistic if sampling effects are quantitatively important. Sampling effects would include the nature of the sample (feces, meat), as well as time and place. For example, the dominant *Campylobacter* types found in processed carcasses have been shown to differ from those found in live chickens entering the processing plant (*19*). Nonetheless, we have shown that easily identifiable sampling effects are overwhelmed by the host effect. Moreover, because analysis within the ST-21 complex (Figure) is robust to identified and unidentified sampling effects, we do not believe this to be a major problem.

**Figure:**
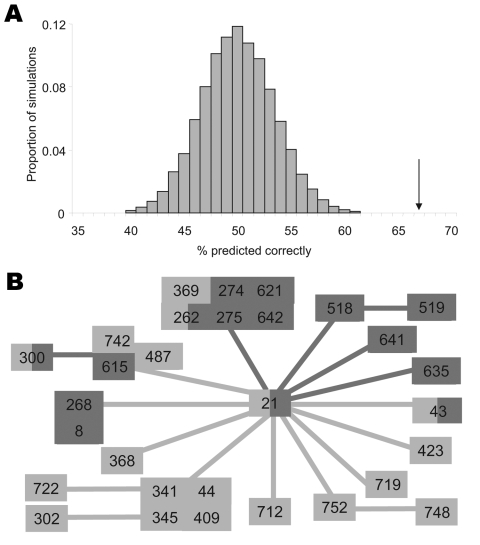
Prediction of source of origin within the sequence type ST-21 complex. A) Observed accuracy of prediction (arrow) compared with distribution of values obtained by permuting host labels so that the alleles varying from central genotype are not informative on host of origin. B) Prediction of origin by using only alleles for which substantial reference information is available. Light lines indicate alleles different from ST-21 present mainly in chickens in the reference population (i.e., an allele that would predict chicken origin); dark lines indicate alleles present mainly in bovids (i.e., predicts bovid origin). Light boxes indicate STs found only in chickens, dark boxes indicate STs found only in bovids, and boxes with light and dark shading indicate STs found in bovids and chickens.

An additional limitation of our allele-based application of STRUCTURE is that it assumes allelic independence, which is clearly violated for the dataset analyzed. Assignment accuracy can be estimated into 2 different ways. The first, which we have used throughout this report, is a holdout procedure whereby source of origin of strains for which the actual origin is known is predicted by using the rest of the sample as a training set. This method provides an unbiased empiric measure of accuracy. To predict isolates for which the source is unknown, this procedure is not possible. Thus, it would be desirable to use estimates of accuracy that the algorithm provides. Because STRUCTURE assumes each allele is independent, its estimate of the accuracy with which it estimates the frequency of a particular multilocus genotype frequency is often overconfident. For example, in differentiating chicken isolates from those originating in cattle and sheep, STRUCTURE predicts an accuracy of 91%, but empirically it achieves an average accuracy of 80%. A better estimate of uncertainty would be necessary for predictive purposes. More sophisticated genetic models that reflect dependence among loci should achieve more accurate assignment as well as better estimates of statistical uncertainty.

Despite these limitations, this approach demonstrates the ability to assign isolates probabilistically to populations. When broad reference populations from the full range of possible sources are available, groups of isolates, such as those affecting a human population over a period of time, can be apportioned to their sources. However, precision in the attribution of *C*. *jejuni* may be less than that of *Salmonella*, in which different animal and bird species appear to host more differentiated populations (*3*). Prediction is possible with individual isolates, in some instances to 1 source, although prediction may suggest a range of populations rather than 1 population. For example, 2 of the sequenced *C*. *jejuni* genomes are from known sources, 1 from a chicken (isolate RM-1221) (*20*) and 1 from a human with campylobacteriosis who had drunk raw milk (isolate 81-176) (*17*,*18*). Assigning these isolates on the basis of reference datasets we used in this report predicted their origin as chicken (99% probability) and cattle/sheep (97% probability), respectively.

The broad host range of *C*. *jejuni*, spanning a variety of mammalian, avian, and other species, makes it a good model for studying features that may be informative of the ecology of multihost pathogens. *C*. *jejuni* acquires genome fragments estimated to be a few hundred bp in length (*21*) from of other members of this species. Our analysis of the ST-21 complex shows that isolates in this complex have acquired genetic material prevalent in the population of *Campylobacter* carried by their host species (Figure). This observation implies that there is persistent differentiation in allele frequencies between different host species and that many ST-21 isolates represent lineages that have persisted within the same host species long enough to acquire a substantial number of alleles.

We surveyed 7 loci and found an average of 0.32 host-specific alleles in 81 STs other than ST-21 that were members of ST-21 complex, i.e., ≈5% of the alleles in this analysis. The acquired genes were approximately evenly distributed between these types, with 6 of the 7 loci having >1 import. The MLST loci were chosen because they represent core metabolic functions of *C*. *jejuni* (*10*) and are not obvious candidates for host adaptation. Therefore, we are likely observing the neutral level of acquiring genetic information. Extrapolating linearly from these 7 loci to 1,654 gene-coding sequences in the *C*. *jejuni* genome (*22*) gives an estimate of 76 genes with alleles typical of a particular host species within each ST-21 complex isolate. This estimate is rough because it is based on fairly limited data and recombination and selection at other genes may be different. However, this approximation shows the potential for substantial adaptation to the most recent host by homologous recombination. Homologous recombination may be an important factor in allowing a bacterial species to colonize a wide range of host species stably while adapting to some extent to each host.

In conclusion, a population genetic approach has allowed host assignment for *C*. *jejuni* for which host-specific markers are unavailable but host species populations are differentiated by allele frequency at a range of loci. Host association appears stronger than temporal and geographic effects. Homologous recombination generates a host signature in the *C*. *jejuni* genome and analyses that use this signal have improved accuracy of host prediction. The inherent standardization and portability of sequence typing in combination with the availability of such improved assignment techniques support the application of this approach to clarify aspects of *C*. *jejuni* epidemiology on a global scale and to study other suitable microbes.

## References

[1] Cleaveland S, Laurenson MK, Taylor LH. Diseases of humans and their domestic mammals: pathogen characteristics, host range and the risk of emergence. Philos Trans R Soc Lond B Biol Sci. 2001;356:991–9. 10.1098/rstb.2001.0889 11516377 PMC1088494

[2] Batz MB, Doyle MP, Morris G Jr, Painter J, Singh R, Tauxe RV, Attributing illness to food. Emerg Infect Dis. 2005;11:993–9. 16022770 10.3201/eid1107.040634 PMC3371809

[3] Hald T, Vose D, Wegener HC, Koupeev T. A Bayesian approach to quantify the contribution of animal-food sources to human salmonellosis. Risk Anal. 2004;24:255–69. 10.1111/j.0272-4332.2004.00427.x 15028016

[4] Wegener HC, Hald T, Lo Fo Wong D, Madsen M, Korsgaard H, Bager F, *Salmonella* control programs in Denmark. Emerg Infect Dis. 2003;9:774–80. 12890316 10.3201/eid0907.030024 PMC3023435

[5] Dingle KE, Colles FM, Ure R, Wagenaar JA, Duim B, Bolton FJ, Molecular characterization of *Campylobacter jejuni* clones: a basis for epidemiologic investigation. Emerg Infect Dis. 2002;8:949–55. 12194772 10.3201/eid0809.02-0122 PMC2732546

[6] Rosef O, Kapperud G, Lauwers S, Gondrosen B. Serotyping of *Campylobacter jejuni, Campylobacter coli*, and *Campylobacter laridis* from domestic and wild animals. Appl Environ Microbiol. 1985;49:1507–10. 4015088 10.1128/aem.49.6.1507-1510.1985 PMC241755

[7] Schouls LM, Reulen S, Duim B, Wagenaar JA, Willems RJ, Dingle KE, Comparative genotyping of *Campylobacter jejuni* by amplified fragment length polymorphism, multilocus sequence typing, and short repeat sequencing: strain diversity, host range, and recombination. J Clin Microbiol. 2003;41:15–26. 10.1128/JCM.41.1.15-26.2003 12517820 PMC149617

[8] Siemer BL, Harrington CS, Nielsen EM, Borck B, Nielsen NL, Engberg J, Genetic relatedness among *Campylobacter jejuni* serotyped isolates of diverse origin as determined by numerical analysis of amplified fragment length polymorphism (AFLP) profiles. J Appl Microbiol. 2004;96:795–802. 10.1111/j.1365-2672.2004.02205.x 15012818

[9] Manning G, Dowson CG, Bagnall MC, Ahmed IH, West M, Newell DG. Multilocus sequence typing for comparison of veterinary and human isolates of *Campylobacter jejuni.* Appl Environ Microbiol. 2003;69:6370–9. 10.1128/AEM.69.11.6370-6379.2003 14602588 PMC262249

[10] Dingle KE, Colles FM, Wareing DR, Ure R, Fox AJ, Bolton FE, Multilocus sequence typing system for *Campylobacter jejuni.* J Clin Microbiol. 2001;39:14–23. 10.1128/JCM.39.1.14-23.2001 11136741 PMC87672

[11] French N, Barrigas M, Brown P, Ribiero P, Williams N, Leatherbarrow H, Spatial epidemiology and natural population structure of *Campylobacter jejuni* colonizing a farmland ecosystem. Environ Microbiol. 2005;7:1116–26. 10.1111/j.1462-2920.2005.00782.x 16011749

[12] Hopkins KL, Desai M, Frost JA, Stanley J, Logan JM. Fluorescent amplified fragment length polymorphism genotyping of *Campylobacter jejuni* and *Campylobacter coli* strains and its relationship with host specificity, serotyping, and phage typing. J Clin Microbiol. 2004;42:229–35. 10.1128/JCM.42.1.229-235.2004 14715757 PMC321682

[13] Smith JM, Smith NH, O’Rourke M, Spratt BG. How clonal are bacteria? Proc Natl Acad Sci U S A. 1993;90:4384–8. 10.1073/pnas.90.10.4384 8506277 PMC46515

[14] Maiden MC, Bygraves JA, Feil E, Morelli G, Russell JE, Urwin R, Multilocus sequence typing: a portable approach to the identification of clones within populations of pathogenic microorganisms. Proc Natl Acad Sci U S A. 1998;95:3140–5. 10.1073/pnas.95.6.3140 9501229 PMC19708

[15] Dingle KE, Colles FM, Falush D, Maiden MC. Sequence typing and comparison of population biology of *Campylobacter coli* and *Campylobacter jejuni.* J Clin Microbiol. 2005;43:340–7. 10.1128/JCM.43.1.340-347.2005 15634992 PMC540151

[16] Pritchard JK, Stephens M, Donnelly P. Inference of population structure using multilocus genotype data. Genetics. 2000;155:945–59. 10835412 10.1093/genetics/155.2.945 PMC1461096

[17] Korlath JA, Osterholm MT, Judy LA, Forfang JC, Robinson RA. A point-source outbreak of campylobacteriosis associated with consumption of raw milk. J Infect Dis. 1985;152:592–6. 4031557 10.1093/infdis/152.3.592

[18] Hofreuter D, Tsai J, Watson RO, Novik V, Altman B, Benitez M, Unique features of a highly pathogenic *Campylobacter jejuni* strain. Infect Immun. 2006;74:4694–707. 10.1128/IAI.00210-06 16861657 PMC1539605

[19] Slader J, Domingue G, Jorgensen F, McAlpine K, Owen RJ, Bolton FJ, Impact of transport crate reuse and of catching and processing on *Campylobacter* and *Salmonella* contamination of broiler chickens. Appl Environ Microbiol. 2002;68:713–9. 10.1128/AEM.68.2.713-719.2002 11823211 PMC126660

[20] Fouts DE, Mongodin EF, Mandrell RE, Miller WG, Rasko DA, Ravel J, Major structural differences and novel potential virulence mechanisms from the genomes of multiple *Campylobacter* species. PLoS Biol. 2005;3:e15. 10.1371/journal.pbio.0030015 15660156 PMC539331

[21] Fearnhead P, Smith NG, Barrigas M, Fox A, French N. Analysis of recombination in *Campylobacter jejuni* from MLST population data. J Mol Evol. 2005;61:333–40. 10.1007/s00239-004-0316-0 16044246

[22] Parkhill J, Wren BW, Mungall K, Ketley JM, Churcher C, Basham D, The genome sequence of the food-borne pathogen *Campylobacter jejuni* reveals hypervariable sequences. Nature. 2000;403:665–8. 10.1038/35001088 10688204

